# Identification and Fine-Mapping of a Novel Locus *qSCL2.4* for Resistance to *Sclerotinia sclerotiorum* in Sunflower (*Helianthus annuus*)

**DOI:** 10.3390/plants14243826

**Published:** 2025-12-16

**Authors:** Mingzhu Zhao, Dexing Wang, Dianxiu Song, Xiaohong Liu, Bing Yi, Yuxuan Cao, Jingang Liu, Liangshan Feng

**Affiliations:** 1 Institute of Crop Research, Liaoning Academy of Agricultural Sciences, Shenyang 110161, China; zhaomingzhu23@163.com (M.Z.);; 2 Liaoning Academy of Agricultural Sciences, Shenyang 110161, China

**Keywords:** *Helianthus annuus*, *Sclerotinia sclerotiorum*, QTL mapping, WRKY transcription factor, haplotypes, disease resistance

## Abstract

*Helianthus annuus* L. is one of the major oilseed crops worldwide, and its production is seriously affected by a highly destructive necrotrophic pathogen, *Sclerotinia sclerotiorum* (*S. sclerotiorum*). The use of resistant cultivars is the best control measure via molecular breeding; however, the gene action underlying resistance to this stress is not well-established. Here, we conducted QTL analysis for *S. sclerotiorum* resistance in a recombinant inbred line (RIL) population that were developed from parents with resistant (C6) and susceptible (B728) to the disease. A high-density genetic linkage map with 6059 single nucleotide polymorphism (SNP) markers and a total length of 2763 cM was developed. The lesion length (LL) and the lesion area (LA) in the field, under climate chamber conditions or greenhouse conditions, were assessed following standardized inoculation protocols. A total of 16 major QTL for LL and 12 for LA were detected across three experimental environments, explaining 1.58–32.86% of the phenotypic variation. Of these, a major-effect QTL, *qSCL2.4* on chromosome 2, could explain 30.22% of phenotypic variance with alleles from parent C6 which had more increased resistance to *S. sclerotiorum*. Fine-mapping in the BC_1_F_3_ population narrowed the locus to a 226.7 kb interval. *HaWRKY48*, which encodes a WRKY transcription factor located in this region, was prioritized as the prime candidate gene. Polymorphism analysis of *HaWRKY48* in 138 sunflower accessions revealed eight SNPs defining six haplotypes. Resistance was associated with Hap3 and susceptibility to Hap1/Hap6. These findings advance our understanding of the genetic mechanisms governing sunflower resistance to *S. sclerotiorum* and provide valuable genetic markers for molecular breeding of resistant cultivars.

## 1. Introduction

Sunflower (*Helianthus annuus* L.) has become an important oilseed crop worldwide [1]. However, its production is severely threatened by *S. sclerotiorum*, a necrotrophic fungus that results in *sclerotinia* stem/head rot [2]. Present control of the disease in many crops is based almost only on restricted fungicides applications, but its poor effectiveness along with environmental pressure have actively driven the search for more sustainable measures [3]. Hence, breeding resistant cultivars via modern breeding methods have become the most effective way in various plant species [4,5]. Resistance to *S. sclerotiorum* is a polygenic trait characterized by additive and epistatic interactions, moderate heritability, and significant genotype-environment interactions [6]. QTL mapping has become an important approach for detecting genomic regions that are linked to resistance of *S. sclerotiorum* in sunflower.

QTL studies employing RFLP markers uncovered four leaf resistance loci and two head resistance loci against *S. sclerotiorum* [7]. Bert et al. [8] revealed 11 QTL associated with pathogen resistance through comparative genetic analyses. Micic et al. [9] reported 15 QTL associated with leaf lesions, stem lesions, and fungal growth rates, accounting for 24.4–33.7% of genotypic variance. Micic et al. [10] applied selective genotyping to confirm QTL for midstalk rot resistance. Rönicke et al. [11] mapped three lesion length (LL)-related and two head rot-related QTL by using AFLP and SSR markers, explaining 10.6–17.1% phenotypic variance. Yue et al. [12] identified 16 head rot resistance QTL, each accounting for 8.4–34.5% phenotypic variation. Amouzadeh et al. [13] localized five resistance QTL to linkage groups 1, 3, 8, 10, and 17 using enhanced SSR/SNP maps.

High-throughput genotyping advancements facilitated more precise QTL detection. Zubrzycki et al. [14] utilized a 384-SNP array to discover 36 major-effect and 13 epistatic QTL. Talukder et al. [15] employed genotyping-by-sequencing to construct a genetic linkage map with 1053 SNP, detecting two major-effect QTL explaining 31.6% and 20.2% variance, respectively. Talukder et al. [16] established a genetic linkage map with 6315 SNP/InDel, identifying 16 head rot resistance QTL across chromosomes 1, 2, 10, 12, 13, 14, 16, and 17. Talukder et al. [17,18] identified 16 basal stalk rot QTL introgressed from *H. praecox* and 14 QTL from *H. petiolaris*, with wild species alleles enhancing resistance at 11 loci. Association mapping efforts also have implicated candidate genes such as *HaCOI1-1*, *HaCOI1-2*, *HaRIC_B*, and *GLP15* in resistance mechanisms [19,20,21,22]. Despite these significant advancements, the genetic mechanisms underlying resistance to *S. sclerotiorum* in sunflower remain poorly understood, particularly at the level of fine-mapped loci and candidate gene validation.

In our previous study, two contrasting sunflower genotypes, disease susceptible B728 and disease resistant C6 were used for a comparative transcriptomic study to investigate the defense responses to *S. sclerotiorum* [23]. The objectives in this study were (1) to identify QTL for *S. sclerotiorum* resistance using a population of RIL derived from a cross between B728 and C6 across three experiments, (2) to narrow the genomic location of the major-effect QTL using BC_1_F_3_ populations, (3) to locate its associated candidate gene, and (4) to determine the natural variation in candidate gene affecting *S. sclerotiorum* resistance in sunflower. These findings will enhance our knowledge of *S. sclerotiorum* resistance mechanisms and support future efforts in molecular breeding to improve disease resistance in sunflowers.

## 2. Materials and Methods

### 2.1. Plant Materials

Two parental oilseed sunflower inbred lines, disease-susceptible genotype B728 and disease-resistant genotype C6 [23], which had been developed by Liaoning Academy of Agricultural Sciences, were used in this study. B728 originated from a natural mutation event in the elite restorer line F150R. C6 was developed from a cross between the maintainer line HA89B and the local landrace Sandaomei. The two parental lines are similar in major agronomic traits such as plant height and flowering time, minimizing the confounding effects of developmental differences on disease phenotyping. A mapping population containing 178 F_5:6_ RILs was developed through the cross between these contrasting parental lines. For fine-mapping the major-effect resistance QTL, a line RIL-5, which combines this QTL inherited from C6 donor parent with predominant genetic similarity to the recurrent parent B728, was selected to backcross with B728 to generate the BC_1_F_3_ population. Additionally, 138 sunflower accessions (Table S1) were utilized to assess allele variations at the candidate gene region identified through QTL analysis. This diverse germplasm collection, including landraces and modern inbred lines, was provided by the Oil Crops Research Institute of the Chinese Academy of Agricultural Sciences (Wuhan, China).

### 2.2. Growth Conditions

Three experiments (field, climate chambers and greenhouse conditions) were utilized to assess disease resistance in this study (Table 1). For 178 RIL and parents, the disease assessment was conducted under field, climate chambers and greenhouse conditions, respectively. For fine-mapping, a total of 3133 individuals from BC_1_F_3_ populations were conducted for the disease assessment under climate chambers condition. For 138 sunflower germplasm, the experiments were conducted under field and climate chambers conditions.

In experiment 1 (Exp1), sunflowers were planted at Shenyang (123.5° E, 41.8° N) in Liaoning Province under field conditions on 15 June 2022. The experiment followed a randomized block design with three repetitions. Each plot was 6 m^2^ and consisted of 20 plants, with seedlings spaced 60 cm spacing between plants and 50 cm between rows. Each plot consisted of 20 plants. Protection lines were consistently set up across the field, following local sunflower production standards for field management. No fungicides were applied throughout the growing season to allow natural disease development.

In experiment 2 (Exp2), seedlings were cultivated in climate chambers using 3 L plastic pots filled with a sterilized vermiculite-nutrient soil mixture, with each genotype having three pots (one plant per pot). Growth parameters were maintained at 28 °C/25 °C (day/night), 70% relative humidity, and 16 h photoperiod under 4000 Lux illumination.

In experiment 3 (Exp3), pot-culture trials were carried out under greenhouse conditions at the Liaoning Academy of Agricultural Sciences in Shenyang (123.543145° E, 41.824543° N), Liaoning Province, during March. Sunflower seedlings were cultivated in 30 × 30 × 35 cm pots containing 18.0 kg field soil per container, arranged in randomized complete blocks with three pots per genotype (three plants per pot).

### 2.3. Pathogen Inoculation and Disease Assessment

*Sclerotinia sclerotiorum* strains were obtained from symptomatic inflorescences of sunflower (*Helianthus annuus*) plants cultivated under field conditions in Shenyang, Liaoning Province, China [23]. Infected tissue segments were transferred onto potato dextrose agar (PDA; BD Difco^TM^, Franklin Lakes, NJ, USA) plates for fungal isolation, and purification was achieved through hyphal tip culture. A representative strain (MQJH, Figure S1) was selected and preserved on PDA slants at 4 °C for long-term storage. For experimental use, a 5 mm mycelial disk was aseptically excised from the actively growing margin of a 5-day-old PDA culture and transferred to fresh PDA medium. To ensure colony uniformity, ten peripheral mycelial plugs were subcultured onto new PDA plates under identical incubation parameters.

For RILs and germplasm, a modified stem inoculation protocol [24] was used to assess sunflower resistance to *S. sclerotiorum* under controlled conditions. At the 4-leaf developmental stage, the third stem internode was surface-disinfected with 75% ethanol and air-dried. A hypodermic needle (23-gauge) with a depth-limiting collar was used to create a standardized wound (2.0 ± 0.2 mm depth) at the center of the third internode. A mycelial plug was positioned adaxial side down onto the wound to ensure direct hyphal contact with vascular tissues. The inoculation site was immediately sealed with two layers of Parafilm to maintain humidity and prevent contamination. Lesion length (LL) was measured longitudinally along the stem axis at 3 days post-inoculation (dpi) using a digital caliper. LL was measured in vivo on intact plants at 3 dpi, without detaching the stems. For each line, three individual plants were inoculated, and LL was measured on each plant.

For RILs and germplasm, a standardized detached leaf assay was also conducted following modified protocols [24]. Fully expanded sunflower leaves at the seedling stage (V4–V6) were excised from healthy plants and surface-sterilized with 70% ethanol for 30 s, followed by three rinses in sterile distilled water. Leaves were placed abaxial side up on moistened filter paper in 90 mm Petri dishes. For inoculation, two 5 mm mycelial plugs from actively growing *S. sclerotiorum* colonies were symmetrically positioned on each leaf, 10 mm apart from the central vein. In addition, for the BC_1_F_3_ populations, the hyphal suspensions were also prepared by homogenizing 5-day-old mycelia in sterile 0.05% Tween-20 solution (*w*/*v*) using a tissue grinder, followed by filtration through four layers of sterile cheesecloth. The suspension was adjusted to a final concentration of 1 × 10^5^ hyphal fragments/mL using a hemocytometer. Each leaf was inoculated with 10 μL of hyphal suspension at four equidistant points along the midvein. Petri dishes were sealed with Parafilm and incubated at 22 ± 1 °C under a 14-h photoperiod (4000 Lux). Lesion area (cm^2^) was measured at 3 dpi using ImageJ v1.53 software with threshold-based segmentation (RGB: 0–120 for necrosis). For the detached leaf assay, three leaves from different plants of each RIL line were inoculated, and the lesion area was measured.

### 2.4. Phenotypic Data Analyses

Statistical analyses were conducted using SPSS Statistics 19.0 for correlation coefficient calculations. Genetic variance partitioning was performed via the Analysis of Variance (ANOVA) module in SPSS 19.0. Broad-sense heritability (*H*2) for each trait was estimated using variance components derived from the ANOVA output according to the following mixed linear model: *H*2 = *σg*^2^/(*σ_g_*^2^ + *σ_ge_*^2^/*e* + *σ_ε_*^2^/*re*), where *σ_g_*^2^ is (*MS_f_* − *MS_fe_*)/*re*, *σg_e_*^2^ is (*MS_fe_* − *MS_e_*)/*r* and *σ_ε_*^2^ is *MS_e_*; *σ_g_*^2^ = genetic variance, *σ_ge_*^2^ = genotype × environment interaction variance, *σ_ε_*^2^ = error variance. *MS_f_*, *MS_fe_* and *MS_e_* represent mean squares of genotype, genotype × environment interaction, and residual error, respectively. *r* = number of biological replicates (*n* = 3), and *e* = number of environments (*e* = 3: field, climate chamber, greenhouse).

### 2.5. Genotyping

DNA was extracted from leaf samples using CTAB method [25]. A high-resolution SNP genotyping platform was established through Specific-Locus Amplified Fragment sequencing (SLAF-seq) across the 178 RILs and parental lines [26]. Enzymatic digestion with RsaI/HaeIII (New England Biolabs, Ipswich, MA, USA) generated cohesive ends, followed by 3′-adenine overhang addition and dual-index adaptor ligation [27] for multiplexed library construction. SLAF libraries were size-selected (300–500 bp) and validated using an Agilent 2100 Bioanalyzer with DNA 1000 chips. Quantitative PCR (Kapa Biosystems, Wilmington, MA, USA) ensured optimal cluster density prior to paired-end sequencing (2 × 100 bp) on the Illumina HiSeq 2500 platform. Technical controls included parallel processing of *Oryza sativa* ssp. *japonica* cv. Nipponbare libraries, leveraging established rice genome annotations (MSU RGAP) for cross-species sequencing fidelity verification.

Quality-filtered sequencing reads were aligned to the *Helianthus annuus* reference genome (HA412.v2.0) using BWA-MEM (v0.7.17) with optimized parameters [28]. Sequence fragments were systematically classified into polymorphic, monomorphic, or repetitive categories through integrative analysis of allele frequency spectra and sequence divergence patterns. SNP calling was performed through parallel processing with GATK HaplotypeCaller (v4.2.6.1) and SAMtools mpileup (v1.15.1), retaining only concordant variants [29]. Only consensus SNPs that were concordant across both callers were retained, followed by rigorous filtering criteria (minor allele frequency [MAF] > 0.05; missing rate < 20%) [30]. Sequencing reproducibility was rigorously verified through technical replicates of 10% randomly chosen samples, demonstrating exceptional consistency (Pearson’s correlation coefficient *r* > 0.98, *p* < 1 × 10^−15^). This stringent quality control framework ensured high-confidence variant datasets for downstream analyses. After quality control, a total of 6059 high-confidence SNPs were retained for genetic map construction.

### 2.6. Linkage Mapping and QTL Analysis

QTL IciMapping 4.2 software [31] was utilized to construct genetic maps, using a permutation test with 1000 iterations at 0.05. The permutation analysis simulates the null distribution of no QTL effect by shuffling phenotypic data relative to genotypic data, thereby providing a stringent significance threshold (LOD ≥ 2.5) that inherently accounts for multiple testing issues. This approach avoids the over-conservative adjustment of Bonferroni correction while maintaining statistical rigor. QTL were named using abbreviated trait names starting with “*q*”, followed by chromosome number or location designating multiple QTL on the same chromosome.

### 2.7. Development of KASP Markers

For fine-mapping, high-resolution genotyping was performed using kompetitive allele-specific PCR (KASP) markers. SNPs within the QTL interval, identified through whole-genome resequencing of parental lines (B728 and C6), were converted into 11 KASP markers (Table S2) for precise genotyping of 3133 BC_1_F_3_ individuals. All reactions were implemented on a 1536-well high-throughput genotyping platform (LGC Genomics, Hertfordshire, UK) following manufacturer specifications. Fluorescence signals were quantified using a Synergy H1 hybrid multimode microplate reader (BMG Labtech, Ortenberg, Germany) with excitation/emission filters optimized for FAM^TM^ and HEX^TM^ dyes.

### 2.8. RNA Isolation and qPCR Analysis

To investigate dynamic transcriptional responses to *S. sclerotiorum* infection, leaf tissues bordering lesion margins were collected from inoculated and mock-inoculated seedlings of B728 and C6 at five time points (0, 12, 24, 36, and 48 hpi) under climate chambers conditions. Total RNA was reverse-transcribed into cDNA using the PrimeScriptRT Reverse Transcription Kit with genomic DNA removal (TaKaRa, Kusatsu, Shiga, Japan). Quantitative real-time PCR (qRT-PCR) was performed on a LightCycler 480 II system (Vazyme Biotech, Nanjing, China) with gene-specific primers. The primer sequences for *LOC110907968* were as follows: F: 5′-ATGTTAAATTATCAGGACGA-3′, R: 5′-TTCGACTCGCTTCTTCACTCCA-3′ (amplicon size: 477 bp). The primer sequences for *LOC110906338* were as follows: F: 5′-GAGAGTTGGGTGCATCCTGT-3′, R: 5′-CCCCTTCCAGTTTTCTCACCC-3′ (amplicon size: 302 bp). The actin (LOC110903735) reference gene primers were as follows: F: 5′-GGAACAGGAATGGTGAAGGC-3′, R: 5′-TCCATGTCATCCCAGTTGCT-3′ (amplicon size: 212 bp). Primer efficiencies were validated to be between 95% and 105%, and melt curve analysis confirmed single amplicons. Each biological replicate comprised three independent plants, with four technical replicates per sample to ensure measurement precision. Relative gene expression levels were normalized to actin and calculated using the 2^−ΔΔCT^.

### 2.9. Detection of HaWRKY48 Gene Polymorphism in Sunflower Germplasm

To analyze the polymorphism of the *HaWRKY48* gene (*LOC110907968*) in 138 sunflower accessions (Table S1) and the parental lines (susceptible B728 and resistant C6), genomic DNA was extracted using the optimized CTAB method [25]. Gene-specific primers (F:TATTACAACTATCCAAAAGGGCCT, R:TGTTTCTAATTACCTAAAAATAGGCA) were used to amplify the full functional region of *HaWRKY48* (including 5′-UTR, exons, introns, and 3′-UTR) in a 50 μL PCR system (containing 25 μL 2× Taq Plus Master Mix, 2 μL each primer, 1 μL genomic DNA, and 20 μL sterile water). Amplicons were verified by 1.2% agarose gel electrophoresis, purified using the E.Z.N.A.^®^ Gel Extraction Kit (Omega Bio-tek, Norcross, GA, USA), and quantified with a NanoDrop 2000 (Thermo Fisher Scientific, Wilmington, MA, USA); purified products were then pooled with dual-index barcodes and sent to BGI (Beijing Genomics Institute, Shenzhen, China) for short-read sequencing on the Illumina HiSeq 2500 platform (Illumina, San Diego, CA, USA).

## 3. Results

### 3.1. Phenotypic Variation in S. sclerotiorum Resistance Across the RIL Population

**Figure 1:**
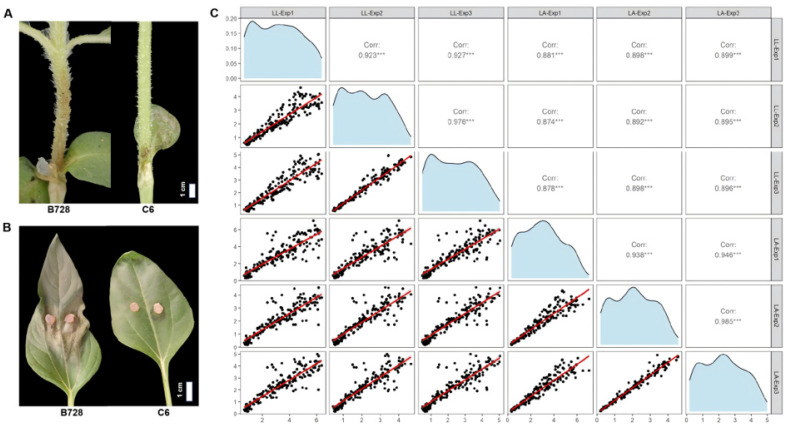
Difference in lesion length (LL) and lesion area (LA) between B728 and C6 (**A**,**B**) and the frequency distribution and Pearson’s correlation coefficients of LL and LA in the RIL population in three environments (**C**). Frequency distribution and Pearson’s correlation analysis of LL and LA in the RIL population across three environments (Exp1: Field, Exp2: Climate chamber, Exp3: Greenhouse). The diagonal panels show the frequency distribution for each trait in each environment, with the x-axis representing the phenotypic value (cm for LL; cm^2^ for LA) and the y-axis representing the number of RILs. The scatter plots below the diagonal display the correlation between traits and environments, with the x-axis and y-axis of each plot corresponding to the respective traits/environments labeled on the diagonal. Above the diagonal line are the correlation coefficient and significant deference. *** represents significant difference at *p* < 0.001.

After infection with *S. sclerotiorum*, the resistant genotype C6 exhibited significantly reduced LL and LA relative to the susceptible B728 (Table 2; Figure 1A,B). The RIL population derived from a cross between C6 and B728 was phenotyped for LL and LA across three independent experimental conditions at 3 dpi (Table S3). Frequency distribution analysis revealed continuous variation in both traits across the RIL population (Figure 1C). LL ranged from 0.42 to 7.15 cm, while LA spanned 0.16 to 7.32 cm^2^. The phenotypic distributions of both LL and LA conformed to normal distribution assumptions, a hallmark of polygenic inheritance controlled by multiple QTL. Correlation analysis between LL and LA revealed a significant positive correlation across all three environments (Figure 1C). In addition to the correlation between traits, the phenotypic values of LL and LA also showed significant positive correlations across different environments. Broad-sense heritability values were 87.6% for LL and 89.3% for LA (Table 2). These high heritability values indicate that the observed phenotypic variation in LL and LA is predominantly driven by genetic factors rather than environmental variation, providing strong justification for subsequent QTL mapping analyses.

### 3.2. High-Density Linkage Mapping and Multi-Environment QTL Detection

Following stringent quality control, 6059 high-confidence SNPs were selected to construct a genome-wide linkage map spanning 2763 cM across 17 chromosomes, with marker density ranging from 157 (Chr8) to 658 (Chr2) loci per chromosome and an average interval of 0.52 cM (Figure S1).

**Figure 2:**
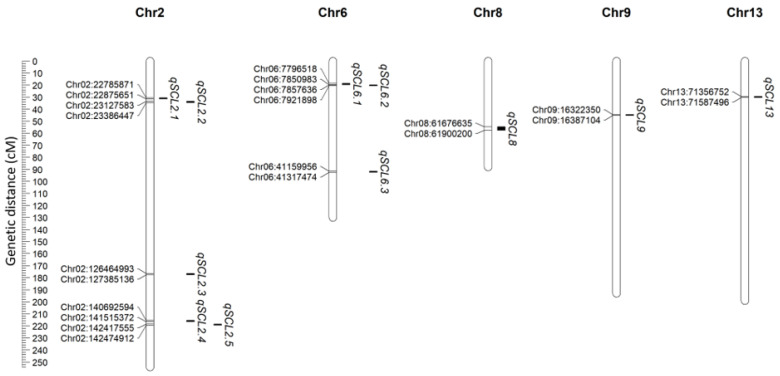
High-resolution genetic linkage map illustrating QTL governing lesion length and lesion area in RIL population. Black annotations denoting novel loci discovered in this investigation. Chromosomal positions reflect recombination frequencies calculated.

Using inclusive composite interval mapping (ICIM) with additive effects model, we detected 11 resistance-associated genomic regions distributed across chromosomes 2, 6, 8, 9, and 13 (Figure 2). Across three environments, we identified 16 QTL for LL and 12 QTL for LA, each of these QTL explains between 1.58 and 32.86% of the observed phenotypic variation in the RIL population (Table 3). Among the 11 resistance-associated genomic regions identified, *qSCL2.4* represents a novel locus not previously reported. which was identified as a stable major-effect locus detected in all environments, contributing 23.39–28.15% and 25.62–32.86% of total variance of LL and LA, respectively. The *qSCL2.4* had allele conferring increased *S. sclerotiorum* resistance derived from the parent C6.

### 3.3. Fine-Mapping of qSCL2.4 and Analyzing Candidate Genes

**Figure 3:**
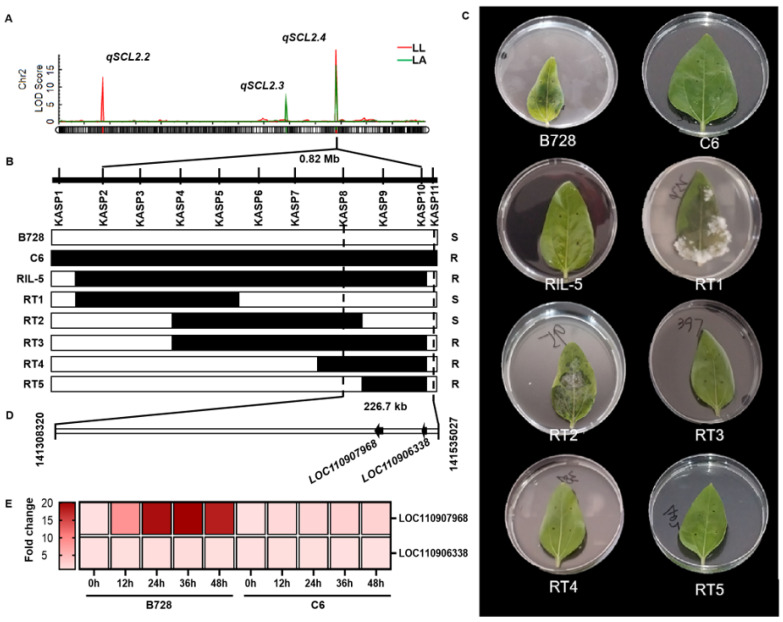
Molecular dissection of *qSCL2.4* locus and functional characterization of associated genes. (**A**) Physical map of the 226.7 kb target interval delineated by flanking molecular markers. (**B**) High-resolution recombination breakpoint analysis using five informative recombinants. Genotypic configurations are depicted as white (B728) and black (C6) haplotype blocks, with vertical dashed lines demarcating the critical candidate region. (**C**) Comparative necrotic lesion development at 3 days post-inoculation (dpi) across recombinant genotypes. (**D**) Genomic architecture of the refined interval showing two annotated open reading frames, with arrow directionality indicating transcriptional orientation. (**E**) qRT-PCR expression levels for two annotated genes at different times post-inoculation.

The major-effect QTL *qSCL2.4* was in an approximate 0.82 Mb interval with flanking by Chr2:140692594 and Chr2:141515372 on chromosome 2 (Figure 3A). To precisely locate this QTL within the genome, resistant line RIL-5 was selected for backcrossing with recipient parent B728 due to their differing alleles at the *qSCL2.4* locus while sharing 71% of genetic background. A total of 3133 offspring from the BC_1_F_3_ generation were genotyped using eleven KASP markers across the target region, identifying five key informative recombinants (Figure 3B). At 3 dpi of *S. sclerotiorum* inoculation, the visible lesions appeared in RT1 and RT2 rather than in RT3, RT4 and RT5 (Figure 3C). Finally, the genomic interval of *qSCL2.4* was reduced from ~0.82 Mb to a 226.7 kb interval flanked by markers KASP8 (Chr2: 141308320) and KASP11 (Chr2: 141535027).

According to the *Helianthus annuus* reference genome *HanXRQr1.0*, this region contains two annotated genes, *LOC110907968* and *LOC110906338*. Of these, *LOC110906338* encodes an unknown protein, while *LOC110907968* encodes WRKY transcription factor 48 (HaWRKY48) (Figure 3D). Following an inoculation with *S. sclerotiorum*, there was no significant change in the expression level of gene *LOC110906338* between B728 and C6, while the expression level of *LOC110907968* was significantly upregulated, especially in B728 (Figure 3E). In conclusion, *LOC110907968* encodes the transcription factor HaWRKY48 and was the candidate gene for *qSCL2.4* in sunflower.

### 3.4. Natural Variation in HaWRKY48 Affects S. sclerotiorum Resistance in Sunflower

**Figure 4:**
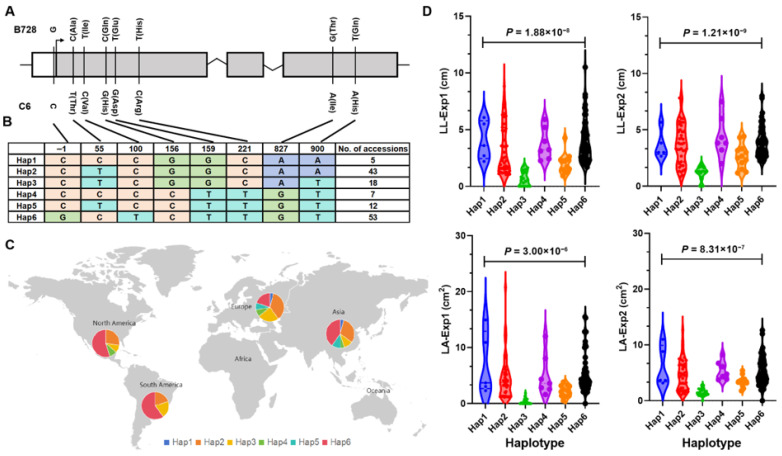
Haplotype analysis for *HaWRKY48* locus in the sunflower accessions. (**A**) Allelic variation in *HaWRKY48* between B728 and C6. (**B**) Six haplotypes for *HaWRKY48* in 138 sunflower accessions. (**C**) Geographic distribution of six haplotypes across four major sunflower-growing regions. (**D**) Evaluation of lesion length (LL) and lesion area (LA) of six haplotypes under two independent experimental conditions at 3 dpi with *S. sclerotiorum*.

We characterized the natural variation in *HaWRKY48* between the parental lines B728 and C6. Sequence alignment revealed one SNP within 5′-UTR and seven non-synonymous SNPs within the exon of *HaWRKY48* (Figure 4A). We genotyped a diverse panel of sunflower using the eight identified SNPs and classified the population into six distinct haplotypes, designated as Hap1, Hap2, Hap3, Hap4, Hap5, and Hap6. In two parental lines, C6 belongs to Hap2, while B728 belongs to Hap6. The frequency of each haplotype varied considerably within the population: Hap6 was the most abundant, with 53 individuals, followed by Hap2 (43 individuals), Hap3 (18 individuals), Hap5 (12 individuals), Hap4 (7 individuals), and Hap1 (5 individuals), respectively (Figure 4B).

We further investigated the geographic distribution of these haplotypes across North America, South America, Europe, and Asia (Figure 4C). Hap6 exhibited the highest frequency in North America (54.55%), South America (60.00%) and Asia (40.21%), while Hap2 was the most prevalent in Europe (36.00%). Hap1 and Hap5 were absent in North America and South America, while Hap4 was not detected in South America. Hap3 displayed a relatively uniform distribution across the four regions, with frequencies ranging from 9.09% to 24.00%. The results suggested that *HaWRKY48* has undergone differential selection pressures during sunflower domestication or adaptation to local environments.

To assess the association between *HaWRKY48* haplotypes and *S. sclerotiorum* resistance, we evaluated LL and LA in the germplasm panel under two experiments at 3 dpi with *S. sclerotiorum* (Figure 4D, Table S1). Significant differences in LL and LA were observed among the six haplotypes. Hap3 consistently exhibited the smallest LL and LA across both environments, which was strongly associated with enhanced resistance. In contrast, Hap1 and Hap6 showed the most severe disease symptoms, with LL and LA values significantly higher than the population average in both environments. Hap2, Hap4, and Hap5 were associated with a broad spectrum of resistance levels. While their average phenotypes were intermediate, Hap2 and Hap4, in particular, showed wide variation in lesion length. These findings not only prioritized the role of *HaWRKY48* as the prime candidate gene for the major-effect QTL *qSCL2.4* but also provided valuable genetic markers for marker-assisted selection in sunflower breeding programs aimed at improving resistance to *S. sclerotiorum* resistance.

## 4. Discussion

### 4.1. Phenotypic of Sunflower Resistance to S. sclerotiorum

Phenotypic evaluation of disease resistance is the foundation of genetic mapping, and the accuracy of trait measurement directly determines the reliability of subsequent QTL identification. In this study, the resistant parent C6 consistently exhibited significantly smaller LL and LA than the susceptible parent B728. The frequency distribution of LL and LA in the RIL population showed continuous variation, which is a typical feature of quantitative traits controlled by multiple genes. This is consistent with the results of earlier studies on sunflower resistance to *S. sclerotiorum* [12,14]. The high heritability of LL and LA in the RIL population indicates that the phenotypic variation is mainly controlled by genetic factors, which is conducive to the detection of major-effect QTL and improves the accuracy and reliability of mapping results. In addition, the positive correlation between LL and LA indicates that they effectively reflect the same resistance mechanism, as the pathogen expands longitudinally along the stem or laterally on the leaf while maintaining a relatively consistent necrotic region morphology. Such consistency between the two traits reduces redundancy in phenotypic evaluation and confirms that either trait can be used as a reliable indicator for resistance screening, though combining both traits improves the accuracy of phenotypic characterization.

### 4.2. Identification of the Novel Locus qSCL2.4

QTL mapping is an effective method to dissect the genetic basis of quantitative traits, and the construction of a high-density genetic map is a prerequisite for accurate QTL localization. In this study, 6059 high-confidence SNPs were used to construct a genome-wide linkage map spanning 2763 cM across 17 chromosomes, with an average marker interval of 0.52 cM. This map has a higher marker density than most previous sunflower genetic maps related to *S. sclerotiorum* resistance [14,15]. The high marker density in this study reduces the possibility of missing QTL and improves the resolution of QTL localization, laying a solid foundation for the identification of the major-effect QTL *qSCL2.4*.

A total of 11 resistance-associated genomic regions were detected across chromosomes 2, 6, 8, 9, and 13, including 16 QTL for LL and 12 QTL for LA. Among these, *qSCL2.4* on chromosome 2 was identified as a stable major-effect QTL, explaining 23.39–28.15% of the phenotypic variation for LL and 25.62–32.86% for LA across all three environments. This is one of the few major-effect QTL for sunflower resistance to *S. sclerotiorum* reported so far. Most previous studies have identified QTL with relatively small effects. For example, Yue et al. [12] detected 16 QTL for head rot resistance, each explaining 8.4–34.5% of the phenotypic variation, with only one QTL (*QDs10d*) explaining more than 30% of the variation; Amouzadeh et al. [13] identified five QTL for basal stem rot resistance, each accounting for 6.4–23.0% of the phenotypic variation. The large effect and environmental stability of *qSCL2.4* make it an ideal target for fine mapping and candidate gene cloning.

To further narrow down the interval of *qSCL2.4*, fine mapping was performed using a BC_1_F_3_ population consisting of 3133 individuals. The QTL interval was successfully reduced from ~0.82 Mb to 226.7 kb using 11 KASP markers. This fine-mapping resolution is higher than that of most previous studies. Talukder et al. [15] mapped the major QTL *Qbsr-10.1* to a 6.3 cM interval (57.9–66.5 cM) on chromosome 10, which corresponds to a physical distance of approximately 10 Mb. Zubrzycki et al. [14] fine-mapped the QTL *qDS-10d* to a 6.0 cM interval (265.1–271.1 cM) on chromosome 10, with an estimated physical distance of ~5 Mb. The high-resolution fine mapping in this study reduces the number of candidate genes.

When comparing the location of *qSCL2.4* with previously reported QTL for sunflower resistance to *S. sclerotiorum*, it was found that *qSCL2.4* is a novel QTL. Talukder et al. [16] identified 16 QTL for head rot resistance on chromosomes 1, 2, 10, 12, 13, 14, 16, and 17, but none of them were located in the 141.3–141.5 Mb region of chromosome 2. Yue et al. [12] detected QTL on chromosome 2 (*QDi2* and *QDs2*), but their physical positions are approximately 100–120 Mb, which are far from the interval of *qSCL2.4*. The identification of this novel major-effect QTL enriches the genetic resources for sunflower resistance to *S. sclerotiorum* and provides a new target for molecular breeding.

### 4.3. Involvement of the WRKY Gene Family in Disease Resistance and Prediction of Candidate Genes

The WRKY gene family has been known for its crucial role in plant defense responses against diverse pathogens [32,33,34]. *WRKY15* and *WRKY33* in oilseed rape interact and play important roles in resistance to *Sclerotinia* [35]. Overexpression of *BnWRKY33* in oilseed rape enhances resistance to *S. sclerotiorum* [36], while *WRKY28* in *Brassica napus* has been found to regulate the resistance response, although its function is complex and can sometimes suppress resistance [37]. The *Arabidopsis* ortholog *WRKY48* demonstrates paradoxical regulatory behaviors, where knockout mutants paradoxically exhibit elevated *PR1* expression and improved *Pseudomonas syringae* resistance via salicylic acid pathway potentiation [38]. In this study, the gene *LOC110907968*, encoding WRKY transcription factor 48, was identified as a candidate gene associated with *qSCL2.4*, which aligns with the established role of WRKY transcription factors in plant immunity.

In this study, the significant difference in the expression level of *HaWRKY48* between B728 and C6 upon *S. sclerotiorum* inoculation further supports its role in the resistance mechanism. Contrary to the typical expectation that resistance genes are highly induced in resistant genotypes, we observed a rapid and strong upregulation of *HaWRKY48* in the susceptible B728, but not in the resistant C6. This pattern is reminiscent of some WRKY transcription factors that act as negative regulators of plant immunity or as key nodes in a complex network where timing and amplitude of expression are critical [38]. It is plausible that the B728 allele of *HaWRKY48*, upon induction, either suppresses effective defense pathways or activates processes that inadvertently benefit the necrotrophic pathogen. Conversely, the C6 allele, by maintaining a steady-state level of expression, may either avoid the negative consequences of hyperactivation or constitutively prime downstream defense genes, thereby providing a more robust and measured resistance response.

The sequence characterization of *HaWRKY48* between resistant (C6) and susceptible (B728) lines identified one SNP in the 5′-UTR, which is well-known to regulate gene transcription efficiency by interacting with *cis*-acting elements or *trans*-acting factors. Thus, the SNP in this region may alter *HaWRKY48* expression levels in B728 and C6. Moreover, non-synonymous SNPs directly result in amino acid substitutions, which could potentially disrupt the DNA-binding affinity of WRKY48, reduce its ability to form functional dimers, or alter its transcriptional activation/repression activity toward downstream defense-related genes. These results suggested that *HaWRKY48* in sunflower may also play a significant role in the defense response against *S. sclerotiorum*.

While our evidence strongly implicates *HaWRKY48* as the candidate gene for *qSCL2.4*, the possibility that the adjacent gene *LOC110906338* contributes to the resistance cannot be entirely ruled out. *LOC110906338* is annotated as encoding a protein of unknown function. Our expression analysis revealed no significant induction of LOC110906338 upon *S. sclerotiorum* infection in either the resistant (C6) or susceptible (B728) parent, which diminishes its likelihood as the primary driver of the observed resistance QTL. Future studies, such as gene editing or transgenic complementation, will be crucial to definitively confirm the functional role of *HaWRKY48* and exclude any potential contribution from *LOC110906338*.

### 4.4. Excellent Allelic Variation in HaWRKY48

Previous studies have found the association between haplotypes in candidate genes and disease resistance [39,40,41]. In this study, 138 sunflower inbred lines were genotyped using the eight SNPs, and six haplotypes were identified. Hap3 showed the smallest LL and LA in both environments, indicating that it is an excellent allelic variation associated with enhanced resistance to *S. sclerotiorum*. In contrast, Hap1 and Hap6 showed the largest LL and LA, representing susceptible allelic variations. Interestingly, the haplotype network suggests that the six haplotypes can be grouped into two groups (Hap1–3 and Hap4–6). Within the former group, Hap3 appears to be a derivative of Hap2. This evolutionary relationship might explain their differential roles in disease resistance. Hap2 shows substantial phenotypic variation in resistance, which can be attributed to genetic background effects. C6, as a representative of resistant Hap2 lines, not only harbors the major-effect QTL *qSCL2.4* but also carries beneficial alleles at minor resistance QTL, including *qSCL2.2*, *qSCL2.3*, and *qSCL6.1*, while susceptible Hap2 accessions may lack these favorable loci. In summary, the strong association between the Hap3 haplotype of *HaWRKY48* and resistance across a diverse germplasm panel provides compelling correlative evidence for its role in disease resistance. Although a formal genetic confirmation through complementation test or analysis of segregating populations fixed for different *HaWRKY48* haplotypes would be ideal, the consistent association observed in our germplasm panel underscores the potential of Hap3 as a valuable breeding allele.

The geographic distribution of *HaWRKY48* haplotypes showed obvious regional differences. Hap6 was the dominant haplotype in North America, South America and Asia. Given that the sunflower was domesticated in North America and subsequently spread to Europe and then to the rest of the world, the prevalence of Hap6 in these continents could reflect its early establishment in the founding germplasm that was widely disseminated. In addition to these historical dispersal patterns, the current distribution may also be influenced by the adaptation of sunflower to local environmental conditions and selection pressures during domestication and breeding. Historically, sunflower breeding programs in North America, South America, and Asia have prioritized traits such as high seed oil content and large flower head size over *S. sclerotiorum* resistance, especially in North America where *S. sclerotiorum* outbreaks were sporadic or less severe in the past [42]. Moreover, the trade-offs between disease resistance and agronomic performance cannot be ruled out. Constitutive expression of defense genes may divert resources from growth and reproduction, leading to yield penalties in the absence of disease [43,44]. If Hap3-mediated resistance incurs such a fitness cost, breeders may have unconsciously selected against it in environments where *S. sclerotiorum* pressure is low, while Hap6, which may lack this cost, would be preferentially retained.

The low frequency of the resistant Hap3 across all regions, while concerning from a disease management perspective, also highlights its significant breeding utilization value. Hap3 provides a molecular target for improving *S. sclerotiorum* resistance in sunflower. The eight SNPs defining Hap3 can be converted into KASP markers, enabling efficient MAS in breeding programs. Unlike conventional phenotypic screening for *S. sclerotiorum* resistance, MAS using Hap3-specific markers allows breeders to select resistant individuals at the seedling stage, significantly accelerating the breeding cycle.

## 5. Conclusions

In this study, a high-density genetic map constructed with 6059 SNPs was developed. We identified 16 QTL for LL and 12 QTL for LA after infection with *S. sclerotiorum*. Among these, the novel major-effect QTL *qSCL2.4* was stably detected across all environments, explaining 23.39–28.15% of the phenotypic variation for LL and 25.62–32.86% for LA. Fine-mapping using a BC_1_F_3_ population narrowed *qSCL2.4* to a 226.7 kb interval. By combining with gene expression analysis, *HaWRKY48* was prioritized as the prime candidate gene for this QTL. In 138 sunflower germplasm accessions, six haplotypes were identified for *HaWRKY48*, with Hap3 being significantly associated with enhanced *S. sclerotiorum* resistance and Hap1/Hap6 linked to susceptibility. These findings advance our understanding of the genetic mechanisms underlying sunflower resistance to *S. sclerotiorum* and provide valuable genetic markers to facilitate molecular breeding of disease-resistant sunflower cultivars.

## Figures and Tables

**Table 1:** Plant materials and growth conditions for QTL analysis, fine-mapping and haplotype analysis.

Objective	Conditions (Year)	Plant Materials	Pathogen Inoculation	Disease Assessment
QTL analysis	Exp1, Field (2022), Exp2, Climate chambers (2023), Exp3, Greenhouse (2022)	RILs and parents	Mycelial plugs	LL and LA, 3 dpi
Fine-mapping	Exp2, Climate chambers (2023)	BC_1_F_3_ and parents	Hyphal suspension	LA, 3 dpi
Haplotype analysis	Exp1, Field (2024), Exp2, Climate chambers (2024)	Germplasm	Mycelial plugs	LL and LA, 3 dpi

LL, lesion length; LA, lesion area; RILs, recombinant inbred lines. For QTL analysis, the 178 RILs and two parental lines were planted in three replicates per environment. In Exp1 (field), each RIL line was represented by one row of 10 plants per replicate. In Exp2 (climate chamber) and Exp3 (greenhouse), each RIL line was represented by three pots (one plant per pot) per replicate. For fine-mapping, a total of 3133 BC_1_F_3_ individuals derived from the cross between RIL-5 and B728 were cultivated in Exp2 (climate chamber), with each plant grown in a separate pot.

**Table 2:** Lesion length (LL) and lesion area (LA) of sunflower RIL population in different conditions.

Trait	Condition	B728	C6	Mean	SE	Min	Max	*H*2
LL (cm)	Exp1	7.27	0.77	3.20	1.72	0.61	7.15	0.94
	Exp2	5.05	0.68	2.20	1.16	0.42	4.96	0.95
	Exp3	5.86	0.51	2.40	1.29	0.42	5.65	0.94
	Average	6.06	0.65	2.60	1.47	0.42	7.15	0.87
LA (cm^2^)	Exp1	7.41	0.31	3.01	1.71	0.28	7.32	0.96
	Exp2	5.52	0.21	2.08	1.18	0.16	5.42	0.95
	Exp3	5.91	0.21	2.28	1.31	0.17	5.49	0.94
	Average	6.28	0.24	2.46	1.47	0.16	7.32	0.89

Mean, average value of the trait across the RIL population; SE, standard error of the mean; Min, minimum value observed; Max, maximum value observed; *H*2, broad-sense heritability, estimated from variance components using a mixed linear model. These values were calculated from the phenotypic data collected from three independent experiments.

**Table 3:** QTL for lesion length and area detected in the sunflower RILs by individual environmental analyses.

QTL	Trait	Condition	Chr.	Left Marker	Right Marker	LOD	PVE (%)	Add
*qSCL2.1*	LL	Exp1	2	Chr02:22785871	Chr02:22875651	12.25	6.26	0.56
*qSCL2.2*	LL	Exp1	2	Chr02:23127583	Chr02:23386447	12.06	14.46	0.79
	LL	Mean	2	Chr02:23127583	Chr02:23386447	11.45	14.48	0.60
*qSCL2.3*	LL	Exp2	2	Chr02:126464993	Chr02:127385136	7.37	3.76	0.41
	LA	Exp2	2	Chr02:126464993	Chr02:127385136	7.04	10.11	0.45
	LA	Exp3	2	Chr02:126464993	Chr02:127385136	6.61	10.53	0.48
	LA	Mean	2	Chr02:126464993	Chr02:127385136	10.35	6.32	0.44
*qSCL2.4*	LL	Exp1	2	Chr02:140692594	Chr02:141515372	14.74	23.39	0.91
	LL	Exp2	2	Chr02:140692594	Chr02:141515372	37.92	28.15	1.11
	LL	Exp3	2	Chr02:140692594	Chr02:141515372	16.23	25.71	0.71
	LL	Mean	2	Chr02:140692594	Chr02:141515372	14.85	25.42	0.73
	LA	Exp1	2	Chr02:140692594	Chr02:141515372	19.26	25.62	1.03
	LA	Exp2	2	Chr02:140692594	Chr02:141515372	37.68	32.86	1.00
	LA	Exp3	2	Chr02:140692594	Chr02:141515372	40.39	30.22	1.23
	LA	Mean	2	Chr02:140692594	Chr02:141515372	19.29	27.31	0.82
*qSCL2.5*	LL	Exp2	2	Chr02:142417555	Chr02:142474912	11.93	6.42	−0.54
	LL	Exp3	2	Chr02:142417555	Chr02:142474912	12.07	7.53	−0.48
	LA	Exp2	2	Chr02:142417555	Chr02:142474912	13.14	6.81	−0.59
*qSCL6.1*	LA	Exp2	6	Chr06:7796518	Chr06:7850983	3.77	1.76	0.36
	LA	Exp3	6	Chr06:7796518	Chr06:7850983	3.01	3.98	0.36
	LA	Mean	6	Chr06:7796518	Chr06:7850983	2.91	4.20	0.39
*qSCL6.2*	LA	Exp1	6	Chr06:7857636	Chr06:7921898	3.10	4.18	0.51
*qSCL6.3*	LL	Exp2	6	Chr06:41159956	Chr06:41317474	3.26	1.76	0.23
	LL	Exp3	6	Chr06:41159956	Chr06:41317474	3.55	1.59	0.28
*qSCL8*	LL	Exp2	8	Chr08:61676635	Chr08:61900200	2.99	1.58	−0.22
*qSCL9*	LL	Exp2	9	Chr09:16322350	Chr09:16387104	3.33	1.86	0.25
	LL	Mean	9	Chr09:16322350	Chr09:16387104	2.62	3.03	0.28
*qSCL13*	LL	Exp2	13	Chr13:71356752	Chr13:71587496	4.18	2.29	0.28

LOD, score indicates the significance of the QTL. PVE, the proportion of phenotypic variance accounted for by the QTL. Add, the effect of substituting one allele from the resistant parent (C6) for the susceptible parent (B728) allele; a positive value indicates that the resistance allele is from C6.

## Data Availability

The original contributions presented in the study are included in the article/Supplementary Material, further inquiries can be directed to the corresponding author.

## Supplementary Materials

The following supporting information can be downloaded at https://www.mdpi.com/article/10.3390/plants14243826/s1, Figure S1: The DNA sequences of *Sclerotinia sclerotiorum* used in this study and their BLAST (version 2.13.0) analysis; Figure S2: The 178 RILs derived from B728 and C6 and its genetic linkage maps identified by 6059 polymorphic SNPs between parents for the 17 chromosomes; Figure S3: KASP genotyping of 3133 BC_1_F_3_ individuals using markers KASP8 and KASP11 for alleles at Chr2:141308320 and Chr2:141535027. Table S1: Sunflower accessions used in this study; Table S2: The Kompetitive Allele Specific PCR markers for fine mapping of qSCL2.4; Table S3: Lesion length (LL) and lesion area (LA) of each RIL in different conditions.
