# Supplementary material for: Identification and Fine-Mapping of a Novel Locus qSCL2.4 for Resistance to Sclerotinia sclerotiorum in Sunflower (Helianthus annuus)
Source: Plants (Basel). 2025 Dec 16;14(24):3826. doi: 10.3390/plants14243826 (PMC12736930; doi:10.3390/plants14243826)
Supplement: Supplementary file 1 [file plants-14-03826-s001.zip › plants-3945517-supplementary.pdf]

Sclerotinia sclerotiorum strain MQJH small subunit ribosomal RNA gene, partial sequence; internal transcribed spacer 1, 5.8S ribosomal RNA gene, and internal transcribed spacer 2, complete sequence; and large subunit ribosomal RNA gene, partial sequence

Sequence ID: MW959798.1 Length: 516 Number of Matches: 1

Range 1: 1 to 515 GenBank Graphics ▼ Next Match ▲ Previous Match

Score	Expect	Identities	Gaps	Strand
952 bits(515)	0.0	515/515(100%)	0/515(0%)	Plus/Plus

```
Query  1    CCTGCGGAAGGATCATTACAGAGTTCATGCCCGAAAGGGTAGACCTCCCACCCTTGTGTA  60
            ||||||||||||||||||||||||||||||||||||||||||||||||||||||||||||
Sbjct  1    CCTGCGGAAGGATCATTACAGAGTTCATGCCCGAAAGGGTAGACCTCCCACCCTTGTGTA  60

Query  61   TTATTACTTTGTTGCTTTGGCGAGCTGCTCTTCGGGGCCTTGTATGCTCGCCAGAGAACA  120
            ||||||||||||||||||||||||||||||||||||||||||||||||||||||||||||
Sbjct  61   TTATTACTTTGTTGCTTTGGCGAGCTGCTCTTCGGGGCCTTGTATGCTCGCCAGAGAACA  120

Query  121  TCAAAACTCTTTTTATTAATGTCGTCTGAGTACTATATAATAGTTAAAACTTTCAACAAC  180
            ||||||||||||||||||||||||||||||||||||||||||||||||||||||||||||
Sbjct  121  TCAAAACTCTTTTTATTAATGTCGTCTGAGTACTATATAATAGTTAAAACTTTCAACAAC  180

Query  181  GGATCTCTTGGTTCTGGCATCGATGAAGAACGCAGCGAAATGCGATAAGTAATGTGAATT  240
            ||||||||||||||||||||||||||||||||||||||||||||||||||||||||||||
Sbjct  181  GGATCTCTTGGTTCTGGCATCGATGAAGAACGCAGCGAAATGCGATAAGTAATGTGAATT  240

Query  241  GCAGAATTCAGTGAATCATCGAATCTTTGAACGCACATTGCGCCCCTTGGTATTCCGGGG  300
            ||||||||||||||||||||||||||||||||||||||||||||||||||||||||||||
Sbjct  241  GCAGAATTCAGTGAATCATCGAATCTTTGAACGCACATTGCGCCCCTTGGTATTCCGGGG  300

Query  301  GGCATGCCTGTTCGAGCGTCATTTCAACCCTCAAGCTCAGCTTGGTATTGAGTCCATGTC  360
            ||||||||||||||||||||||||||||||||||||||||||||||||||||||||||||
Sbjct  301  GGCATGCCTGTTCGAGCGTCATTTCAACCCTCAAGCTCAGCTTGGTATTGAGTCCATGTC  360

Query  361  AGTAATGGCAGGCTCTAAAATCAGTGGCGGCGCCGCTGGGTCCTGAACGTAGTAATATCT  420
            ||||||||||||||||||||||||||||||||||||||||||||||||||||||||||||
Sbjct  361  AGTAATGGCAGGCTCTAAAATCAGTGGCGGCGCCGCTGGGTCCTGAACGTAGTAATATCT  420

Query  421  CTCGTTACAGGTTCTCGGTGTGCTTCTGCCAAAACCCAAATTTTCTATGGTTGACCTCGG  480
            ||||||||||||||||||||||||||||||||||||||||||||||||||||||||||||
Sbjct  421  CTCGTTACAGGTTCTCGGTGTGCTTCTGCCAAAACCCAAATTTTCTATGGTTGACCTCGG  480

Query  481  ATCAGGTAGGGATACCCGCTGAACTTAAGCATATC  515
            |||||||||||||||||||||||||||||||||||
Sbjct  481  ATCAGGTAGGGATACCCGCTGAACTTAAGCATATC  515
```

Figure S1 The DNA sequences of Sclerotinia sclerotiorum used in this study and their BLAST analysis.

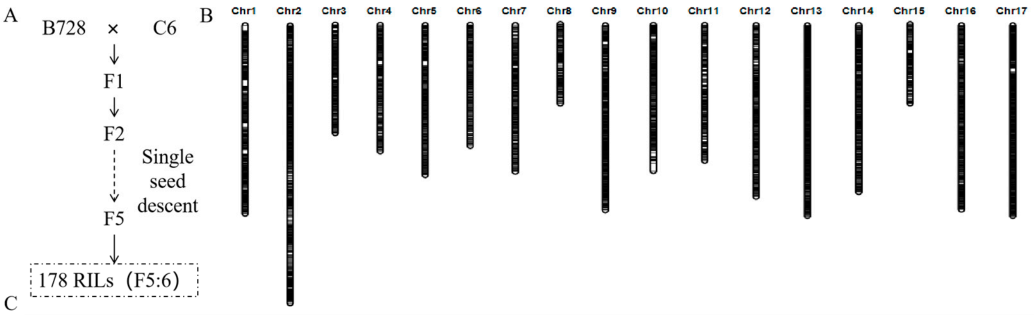

C

	Chr1	Chr2	Chr3	Chr4	Chr5	Chr6	Chr7	Chr8	Chr9	Chr10	Chr11	Chr12	Chr13	Chr14	Chr15	Chr16	Chr17	Total
No.of SNPs	409	658	228	252	330	230	277	157	482	294	246	354	602	396	198	397	549	6059
Linkage map (cM)	196	254	118	135	159	130	155	104	193	155	145	180	199	175	103	192	199	2763
Average marker interval (cM)	0.53	0.42	0.54	0.59	0.52	0.59	0.59	0.62	0.44	0.57	0.61	0.55	0.40	0.49	0.54	0.50	0.38	0.52

Figure S2 The 178 RILs derived from B728 and C6 and its genetic linkage maps identified by 6059 polymorphic SNPs between parents for the 17 chromosomes.

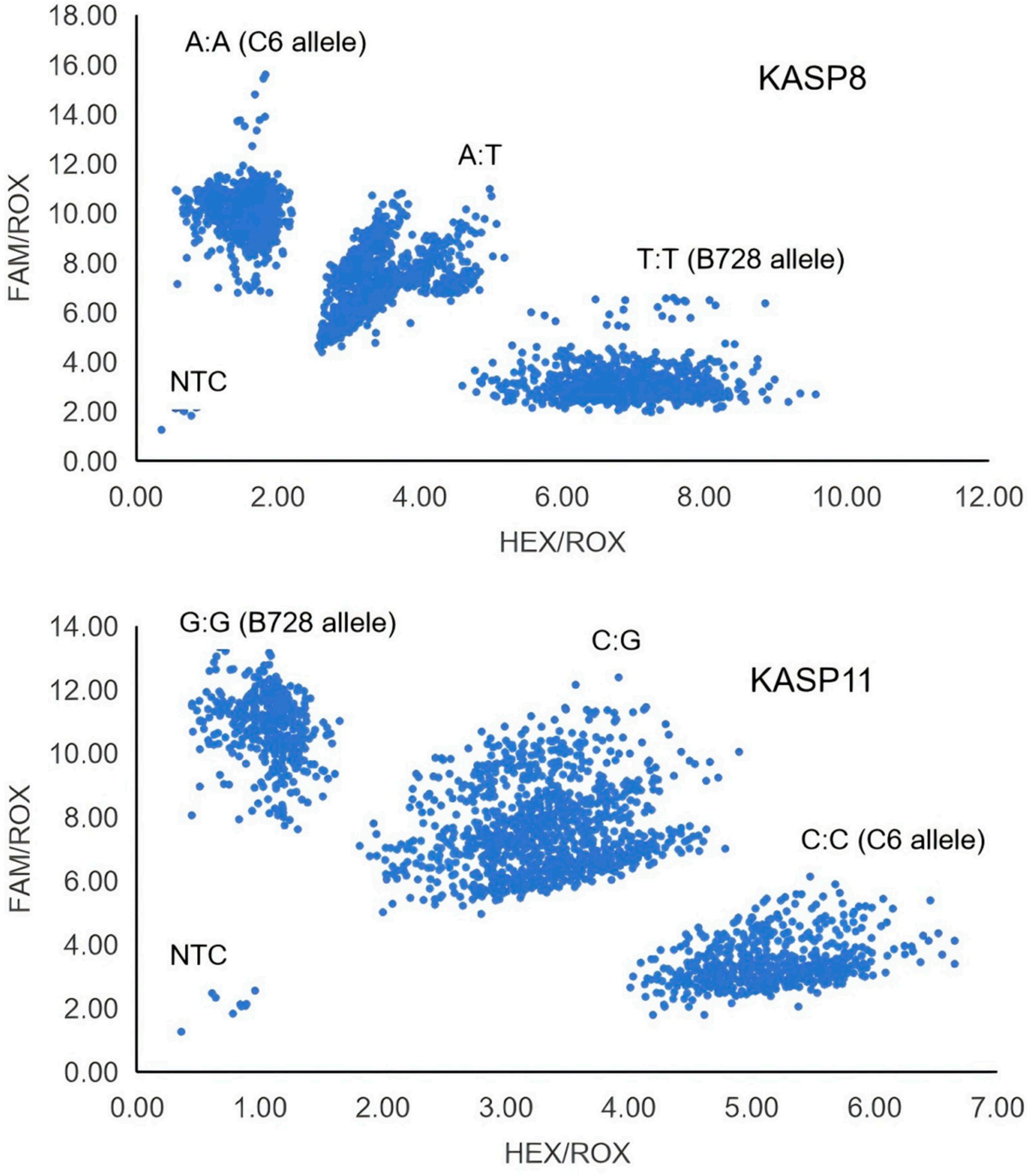

Figure S3 KASP genotyping of 3,133 BC_1F_3 individuals using markers KASP8 and KASP11 for alleles at Chr2:141308320 and Chr2:141535027.

Table S1 Sunflower accessions used in this study and their lesion length (LL) and lesion area (LA) in different conditions.

Accessions	Code	Origin	Haplotypes for *HaWRKY48*	LL-exp1 (cm)	LL-exp2 (cm)	LA-exp1 (cm^2)	LA-exp2 (cm^2)
8523	WXRK0197	South America	Hap6	4.20	4.83	10.53	9.06
8528	WXRK0202	South America	Hap6	1.97	2.33	12.83	7.34
2340841	ZXRK1330	Asia	Hap2	1.07	3.33	1.29	1.75
A27-1	WXRK0201	South America	Hap2	8.83	5.67	11.60	6.27
A27-2	WXRK0201	South America	Hap3	1.17	1.33	0.00	2.67
-	WXRK0091	Europe	Hap2	4.93	7.83	2.15	1.97
118706-1	WXRK0233	North America	Hap6	2.51	2.17	4.82	6.74
118706-2	WXRK0233	North America	Hap2	7.20	5.50	4.06	4.03
1MPIRA	WXRK0107	Europe	Hap5	1.47	2.67	1.25	2.21
2119628-1	ZXRK1328	Asia	Hap6	2.02	2.25	3.45	2.03
2119628-2	ZXRK1329	Asia	Hap5	1.73	4.42	1.25	1.79
76-64A	WXRK0801	Asia	Hap2	1.50	1.33	8.21	9.64
84-14-1-4-1-6A-1	ZXRK1458	Asia	Hap3	0.00	0.00	0.00	1.31
84-14-1-4-1-6A-2	ZXRK1458	Asia	Hap3	0.83	0.83	0.00	1.11
85500-1	WXRK0229	North America	Hap6	3.77	3.00	2.07	3.52
85500-2	WXRK0229	North America	Hap3	0.50	1.17	0.12	1.43
Ba Er Gan-1	WXRK0138	Europe	Hap4	2.27	3.83	1.63	4.78
Ba Er Gan-2	WXRK0138	Europe	Hap2	1.80	4.83	1.24	1.82
Ba Kui-120	ZXRK1847	Asia	Hap3	0.00	1.63	0.00	1.21
Ba Kui-143	ZXRK1868	Asia	Hap6	3.33	2.83	2.24	3.60
Ba Kui-49	ZXRK1809	Asia	Hap2	1.12	0.98	1.25	2.29

Ba Kui-89-1	ZXRK1826	Asia	Hap2	5.10	6.00	20.74	8.50
Ba Kui-89-2	ZXRK1826	Asia	Hap6	2.30	5.25	5.33	7.88
Ba Kui-89-3	ZXRK1826	Asia	Hap6	3.25	3.89	4.35	6.18
Bai Kui Hua	ZXRK0694	Asia	Hap6	4.50	3.83	6.08	4.78
Bai You Kui	ZXRK3015	Asia	Hap6	6.70	3.92	5.12	6.45
Bao 2	WXRK0066	Europe	Hap2	5.50	5.75	8.28	5.02
Ben Lei	ZXRK2964	Asia	Hap6	5.60	4.00	1.85	2.34
Chang Li Xiang-1	ZXRK3276	Asia	Hap1	5.50	3.83	2.32	3.67
Chang Li Xiang-2	ZXRK3276	Asia	Hap6	3.12	3.00	3.21	3.84
Chang Li Xiang-3	ZXRK3276	Asia	Hap6	2.25	3.17	2.35	2.07
Chang Li Xiang-4	ZXRK3276	Asia	Hap6	3.13	3.17	4.12	3.40
Da Hei Gua Zi	ZXRK	Asia	Hap2	6.40	4.25	5.52	6.52
Deng Ta-1	WXRK0109	Europe	Hap6	7.87	7.83	3.30	5.00
Deng Ta-2	WXRK0109	Europe	Hap6	3.00	4.17	0.00	0.00
Deng Ta-3	WXRK0109	Europe	Hap2	1.70	1.67	1.25	1.63
Deng Ta-4	WXRK0109	Europe	Hap3	0.00	0.00	0.71	1.57
Deng Ta-5	WXRK0109	Europe	Hap6	4.13	3.75	3.89	4.63
F(-)-18	WXRK0367	North America	Hap2	6.13	4.17	6.15	4.53
F(-)-20	WXRK0369	North America	Hap6	2.25	2.67	2.69	1.95
F(-)-25	WXRK0374	North America	Hap4	3.23	2.00	3.62	3.85
Fa You	WXRK0116	Europe	Hap3	1.23	1.33	0.00	1.06
Fa You 1-1	WXRK0116	Europe	Hap3	0.00	1.33	0.46	1.17
Fa You 1-2	WXRK0116	Europe	Hap1	2.70	3.00	3.07	3.20
Fu 1-1	WXRK0112	Europe	Hap2	1.60	1.83	1.35	1.28
Fu 1-2	WXRK0112	Europe	Hap3	0.30	1.21	0.00	1.42
Fu 1-3	WXRK0112	Europe	Hap2	6.20	5.38	5.24	6.38

He Qu Kui Hua	ZXRK3343	Asia	Hap2	5.20	3.33	4.65	4.91
He Tao Hei Kui	WXRK0691	Asia	Hap5	2.23	3.33	0.54	3.52
He Tao Hua Mei-1	WXRK0635	Asia	Hap6	2.17	3.00	3.17	4.21
He Tao Hua Mei-2	WXRK0635	Asia	Hap2	3.50	5.67	1.35	1.73
Hei Da Pian Kui Hua Zi-1	ZXRK3259	Asia	Hap6	2.25	4.17	4.11	4.34
Hei Da Pian Kui Hua Zi-2	ZXRK3259	Asia	Hap2	2.05	3.15	4.10	4.99
Hei Jing 2	ZXRK1352	Asia	Hap3	0.00	0.67	0.89	0.94
Hei Ke	WXRK0105	Europe	Hap6	8.10	5.75	5.44	3.30
Ji Kui 119-1	ZXRK1314	Asia	Hap6	2.10	4.00	3.56	5.35
Ji Kui 119-2	ZXRK1314	Asia	Hap2	4.90	5.00	6.01	10.61
Ji Kui 121	ZXRK1316	Asia	Hap4	5.20	6.00	4.36	4.33
Ji Kui 125-1	ZXRK1320	Asia	Hap6	4.90	6.17	2.10	3.06
Ji Kui 125-2	ZXRK1320	Asia	Hap4	5.90	7.50	12.01	8.26
Ji Kui 46-1	ZXRK1241	Asia	Hap6	4.63	3.00	8.28	8.58
Ji Kui 46-2	ZXRK1241	Asia	Hap6	4.65	3.75	2.26	3.36
Ji Kui 49	ZXRK1244	Asia	Hap2	7.47	7.67	2.93	3.14
Ji Kui 6	ZXRK1201	Asia	Hap5	0.93	3.00	2.12	3.62
Ji Kui 62	ZXRK1257	Asia	Hap6	6.93	5.83	6.24	7.05
Ji Kui 64	ZXRK1259	Asia	Hap6	2.70	3.83	10.00	6.63
Ji Kui 81-1	ZXRK1276	Asia	Hap5	4.27	4.33	2.56	3.59
Ji Kui 81-2	ZXRK1276	Asia	Hap2	2.25	3.75	3.25	4.51
Jia Xian Xiang Ri Kui	ZXRK3009	Asia	Hap6	3.53	4.00	3.45	4.16
Jin Xiang You Kui-1	ZXRK3435	Asia	Hap6	2.57	2.25	3.21	4.71
Jin Xiang You Kui-2	ZXRK3435	Asia	Hap6	2.17	3.33	4.28	6.05
Ku Er Le	ZXRK2976	Asia	Hap2	1.90	3.25	4.10	7.76
Kui Hua	ZXRK3261	Asia	Hap3	1.50	1.75	0.00	2.13

Lan Tun Kui Hua	ZXRK1431	Asia	Hap5	1.23	1.18	1.82	2.74
Ling Qiu Bai Kui Hua-1	ZXRK0896	Asia	Hap1	3.60	3.00	10.92	8.81
Ling Qiu Bai Kui Hua-2	ZXRK0896	Asia	Hap6	3.57	3.17	15.30	11.98
Ling Qiu Bai Kui Hua-3	ZXRK0896	Asia	Hap1	2.20	2.67	14.93	10.99
Ling Qiu Hua Li Kui	ZXRK0898	Asia	Hap5	1.53	1.50	2.35	4.82
Ling Qiu Kui Hua	ZXRK0894	Asia	Hap2	2.57	1.83	6.58	7.18
Ma Jia Gang Hei Kui-1	ZXRK2969	Asia	Hap2	2.25	4.21	3.65	4.79
Ma Jia Gang Hei Kui-2	ZXRK2969	Asia	Hap2	3.56	4.50	8.56	12.66
Nong Yuan 87-18	ZXRK1443	Asia	Hap1	6.07	5.67	3.71	3.69
Nong Yuan 87-5-1-1	ZXRK1445	Asia	Hap6	3.50	4.00	4.12	12.62
Nong Yuan 87-5-1-2	ZXRK1445	Asia	Hap2	1.60	0.83	1.12	1.28
Nong Yuan 87-5-2	ZXRK1446	Asia	Hap5	2.35	2.50	3.32	3.88
OH398-1	WXRK0139	Europe	Hap6	4.53	5.83	3.25	4.28
OH398-2	WXRK0139	Europe	Hap3	0.43	1.67	1.32	1.82
OH398-3	WXRK0139	Europe	Hap5	2.63	3.17	1.44	3.20
ohara4a	WXRK0162	South America	Hap6	2.70	2.50	2.91	2.77
P12	WXRK0214	North America	Hap6	2.27	2.67	4.23	5.87
P13	WXRK0215	North America	Hap6	5.60	5.25	4.23	3.45
P4	WXRK0210	North America	Hap2	4.07	3.75	12.21	6.11
P9	WXRK0213	North America	Hap6	3.73	4.33	15.49	8.26
Pi Shan Xiang Ri Kui-1	ZXRK2999	Asia	Hap6	4.77	3.92	3.56	3.71
Pi Shan Xiang Ri Kui-2	ZXRK2999	Asia	Hap6	3.14	2.75	5.62	8.51
Qing An Kui Hua	ZXRK1348	Asia	Hap4	3.12	3.22	2.84	6.72
R018-1	WXRK0238	Europe	Hap3	1.63	1.33	0.80	2.82
R018-2	WXRK0238	Europe	Hap4	3.97	4.33	2.56	3.77
R022	WXRK0239	Europe	Hap2	3.00	2.17	4.12	4.20

S-25-1	WXRK0142	Europe	Hap2	1.17	3.17	1.30	3.25
S-25-2	WXRK0142	Europe	Hap2	1.22	4.83	3.29	4.45
San Dao Bai	ZXRK3270	Asia	Hap2	1.21	2.21	5.09	7.44
San Dao Mei-1	ZXRK0870	Asia	Hap2	5.50	3.83	1.96	3.11
San Dao Mei-2	ZXRK3270	Asia	Hap2	1.26	1.38	1.24	1.01
San Dao Mei-3	ZXRK3295	Asia	Hap6	3.43	3.33	12.80	9.48
San Dao Mei-4	ZXRK3295	Asia	Hap4	2.43	3.83	8.08	6.12
San Dao Mei-5	ZXRK2992	Asia	Hap2	6.97	2.67	3.81	2.15
San Dao Mei-6	ZXRK2992	Asia	Hap3	1.80	2.00	0.00	1.71
San Dao Mei-7	ZXRK3013	Asia	Hap3	1.45	1.25	0.47	0.78
San Dao Mei-8	ZXRK3024	Asia	Hap5	2.33	2.10	3.85	3.33
Tai 2-1	ZXRK1344	Asia	Hap6	10.50	8.00	4.28	2.89
Tai 2-2	ZXRK1344	Asia	Hap6	7.20	4.83	2.14	3.89
Tai He Kui Hua Zi-1	ZXRK3418	Asia	Hap6	2.33	2.67	10.18	7.21
Tai He Kui Hua Zi-2	ZXRK3418	Asia	Hap3	0.60	1.75	0.00	1.13
Te Ke Si You Kui-1	ZXRK3465	Asia	Hap2	3.53	3.25	5.24	5.11
Tong Xian	ZXRK3022	Asia	Hap6	6.33	3.17	3.76	7.46
Tong Xian Ni Gua Zi	ZXRK3022	Asia	Hap6	1.70	2.33	7.29	3.00
Tu Zuo Kui Hua-2-1	ZXRK1412	Asia	Hap2	1.73	2.17	3.24	6.58
Tu Zuo Kui Hua-2-2	ZXRK1412	Asia	Hap2	1.25	3.33	1.35	1.45
Wang Cang Xiang Ri Kui	ZXRK1688	Asia	Hap2	1.36	1.47	1.15	1.79
Wu Chuan Kui Hua-1	ZXRK1432	Asia	Hap3	0.00	0.00	0.00	0.00
WXRK0365	WXRK0365	Asia	Hap2	6.30	6.00	3.25	3.16
Xi Zang Xiang Ri Kui-1	ZXRK3396	Asia	Hap2	1.35	1.25	13.30	7.26
Xi Zang Xiang Ri Kui-2	ZXRK3401	Asia	Hap2	1.57	2.00	3.62	2.29
Xi Zang Xiang Ri Kui-3	ZXRK3401	Asia	Hap6	4.25	4.00	12.77	5.47

Xiang Ri Kui Yi Hao	ZXRK3412	Asia	Hap2	1.03	3.83	4.34	5.71
Xiao Hei San Dao Men	ZXRK3467	Asia	Hap2	3.57	6.17	5.62	7.54
Xin Jiang A Ke	ZXRK2988	Asia	Hap6	1.93	2.67	5.63	6.67
Yan Qi Bai Gua	ZXRK2963	Asia	Hap6	5.70	4.75	3.52	5.19
You Kui-1	ZXRK3255	Asia	Hap5	2.25	2.33	3.11	3.53
You Kui-2	ZXRK3255	Asia	Hap5	1.27	1.25	3.21	5.42
You Kui-3	ZXRK2956	Asia	Hap6	2.70	5.33	4.25	8.07
Zao Shu Bai Mei-1	ZXRK2995	Asia	Hap6	6.35	5.00	5.35	6.92
Zao Shu Bai Mei-2	ZXRK2995	Asia	Hap6	5.10	4.67	3.25	3.63
Zao Shu Bai Mei-3	ZXRK2995	Asia	Hap3	0.70	1.33	1.86	2.22
Zao Shu Quan Hei	ZXRK2954	Asia	Hap6	3.13	6.50	11.06	4.42
Zao Shu Yuan Gua	ZXRK2994	Asia	Hap6	3.25	3.00	6.41	4.69
ZXRK3396	ZXRK3396	Asia	Hap2	2.50	4.83	2.01	1.63

Table S2 The Kompetitive Allele Specific PCR markers for fine mapping of *qSCL2.4*.

Marker	Position	FAM primer(5'-3')	HEX primer(5'-3')	Common primer(5'-3')
KASP 1	Chr02:140579613	GAAGGTGACCAAGTTCATGCTCTTTAATAGAGTTAAAAGTCGGAC	GAAGGTCGGAGTCAACGGATTCTTTAATAGAGTTAAAAGTCGGAT	TGACTGGTCCTAATTTTCATCATAT
KASP 2	Chr02:140692594	GAAGGTGACCAAGTTCATGCTGTTTTTCACTTATGTCATTTATTAT	GAAGGTCGGAGTCAACGGATTGTTTTTCACTTATGTCATTTATTAA	TAAATTTCAACGTGTAGGATGAATT
KASP 3	Chr02:140788182	GAAGGTGACCAAGTTCATGCTGAGAGCTTTATCATAATTAAAAACA	GAAGGTCGGAGTCAACGGATTGAGAGCTTTATCATAATTAAAAACG	CGATTCTTTGCGATCTAACTTCTAGA
KASP 4	Chr02:140889979	GAAGGTGACCAAGTTCATGCTTAAACCGGTACTAAAAGCTCTTGTA	GAAGGTCGGAGTCAACGGATTTAAACCGGTACTAAAAGCTCTTGTG	ATTGATGCGTGTCGAGTGCGATATA
KASP 5	Chr02:140989348	GAAGGTGACCAAGTTCATGCTAGCAATAATCAACTCGCGGGCCTG	GAAGGTCGGAGTCAACGGATTAGCAATAATCAACTCGCGGGCCTC	ATGCCAATGTTGGTTGTGCCAAGAT
KASP 6	Chr02:141089611	GAAGGTGACCAAGTTCATGCTTACATTACATTTATATTTATGTATT	GAAGGTCGGAGTCAACGGATTTACATTACATTTATATTTATGTATA	TAACATGGAATTTTTATACAAATCTC
KASP 7	Chr02:141184895	GAAGGTGACCAAGTTCATGCTAGTTTAAATCTTGACAAGTGTAAT	GAAGGTCGGAGTCAACGGATTAGTTTAAATCTTGACAAGTGTAAA	CAAACCATAGCGATACTACATTCTT
KASP 8	Chr02:141308320	GAAGGTGACCAAGTTCATGCTCTTTTCATATATGATTGGTTCTTTTA	GAAGGTCGGAGTCAACGGATTCTTTTCATATATGATTGGTTCTTTTT	TGTTGCCTTAGACGCCAGTCCTCGA
KASP 9	Chr02:141409173	GAAGGTGACCAAGTTCATGCTATTTTGGGGGTGACAGATTGGTATC	GAAGGTCGGAGTCAACGGATTATTTTGGGGGTGACAGATTGGTATT	GATACCAATCTGTCACCCCCAAAAT
KASP 10	Chr02:141515372	GAAGGTGACCAAGTTCATGCTTCGTGTTGGTTGACGGATCCAAAG	GAAGGTCGGAGTCAACGGATTTCGTGTTGGTTGACGGATCCAAAT	GCTGATCAATCATTCAAAATATAAA
KASP 11	Chr02:141535027	GAAGGTGACCAAGTTCATGCTACAGTTTTGTTTTAGGAAAGTCATG	GAAGGTCGGAGTCAACGGATTACAGTTTTGTTTTAGGAAAGTCATC	TATTAGTTGATAGCGCAATTTGGCTT

Table S3 Lesion length (LL) and lesion area (LA) of each RIL in different conditions.

Line	LL-Exp1	LL-Exp2	LL-Exp3	LL-mean	LA-Exp1	LA-Exp2	LA-Exp3	LLA-mean
RIL-1	3.9105	2.7161	2.8854	3.1707	2.9676	2.8711	3.0370	2.9586
RIL-2	4.3009	3.1397	3.5091	3.6499	3.5773	3.3388	3.4318	3.4493
RIL-3	1.6956	1.1210	1.2401	1.3522	1.3840	0.6944	0.8674	0.9819
RIL-4	1.0483	0.7069	0.7741	0.8431	0.4161	0.3516	0.4005	0.3894
RIL-5	0.8904	0.5652	0.6583	0.7047	0.3391	0.1886	0.1863	0.2380
RIL-6	3.4463	2.6981	2.8719	3.0054	3.3187	2.3064	2.2307	2.6186
RIL-7	4.4209	2.9247	3.1367	3.4941	3.9000	2.4994	2.9405	3.1133
RIL-8	0.8370	0.6694	0.7267	0.7444	0.6288	0.3530	0.5166	0.4994
RIL-9	2.9652	2.2097	2.5186	2.5645	5.7349	3.4151	3.6964	4.2821
RIL-10	2.3162	1.4319	1.5670	1.7717	2.6657	1.5044	1.3554	1.8419
RIL-11	5.5275	3.3210	3.6336	4.1607	5.0749	3.6283	3.1916	3.9649
RIL-12	0.8976	0.8228	0.8778	0.8661	0.9269	0.4650	0.5809	0.6576
RIL-13	0.8640	0.4590	0.5130	0.6120	0.6286	0.5312	0.6051	0.5883
RIL-14	3.0260	1.7862	1.9123	2.2415	2.4622	1.3693	1.3532	1.7282
RIL-15	1.0320	0.9520	0.9690	0.9843	0.9328	0.6483	0.6270	0.7360
RIL-16	2.2671	2.0073	2.0803	2.1182	2.2182	1.7739	1.9260	1.9727
RIL-17	1.5225	0.8987	1.1383	1.1865	1.3253	0.9877	1.1257	1.1463
RIL-18	3.7976	2.3444	2.6478	2.9300	3.5091	2.1693	2.3740	2.6841
RIL-19	3.5267	3.4120	3.6091	3.5159	4.3156	2.5929	2.8369	3.2484
RIL-20	1.6570	1.5465	1.5896	1.5977	1.8015	1.0728	1.1612	1.3452
RIL-21	1.8481	0.9272	1.1582	1.3112	1.8448	1.0411	0.9380	1.2746
RIL-22	1.6220	1.3709	1.5613	1.5181	1.9486	1.3932	1.2255	1.5224
RIL-23	2.7035	1.5035	1.4858	1.8976	4.7769	3.7436	4.1036	4.2080
RIL-24	5.6604	3.9338	3.8047	4.4663	5.0950	3.3706	3.6150	4.0269
RIL-25	1.5522	0.9947	1.1703	1.2391	1.1284	0.9024	0.9798	1.0035
RIL-26	4.1273	2.3167	3.3907	3.2782	3.9878	2.7699	2.9425	3.2334
RIL-27	2.1388	1.2737	1.3786	1.5970	1.8431	1.3455	1.5038	1.5642
RIL-28	2.7979	1.5790	1.4227	1.9332	5.3083	3.5094	3.8822	4.2333
RIL-29	1.2198	0.8721	0.7671	0.9530	1.1636	0.7846	0.8592	0.9358
RIL-30	0.8798	0.6895	0.7558	0.7751	0.7432	0.4718	0.5495	0.5882
RIL-31	2.9200	1.9317	2.0718	2.3078	2.5932	2.0302	2.1610	2.2615
RIL-32	0.7700	0.6158	0.6686	0.6848	0.5276	0.3490	0.3743	0.4170
RIL-33	1.4222	0.9878	1.0494	1.1531	1.2170	0.9732	1.0567	1.0823
RIL-34	2.3520	1.7170	1.9190	1.9960	5.5654	3.4358	3.8805	4.2939
RIL-35	1.6534	1.0931	1.2092	1.3185	1.6003	0.9446	1.0113	1.1854
RIL-36	1.0358	0.6984	0.7648	0.8330	0.5514	0.5086	0.5177	0.5259
RIL-37	1.8017	1.1438	1.3321	1.4259	1.7600	1.5583	1.6150	1.6444
RIL-38	1.3165	1.0307	1.0971	1.1481	1.2326	0.7276	0.9216	0.9606
RIL-39	3.7482	2.4796	2.6594	2.9624	3.4257	2.1149	2.3886	2.6431
RIL-40	1.6359	1.3083	1.4204	1.4548	1.5229	1.4733	1.5585	1.5182
RIL-41	0.9548	0.5895	0.6658	0.7367	0.5138	0.4795	0.4929	0.4954
RIL-42	2.5830	2.4990	2.6434	2.5751	2.6133	1.9078	2.1322	2.2178

RIL-43	4.6380	4.3288	4.4493	4.4720	5.7197	3.7814	4.1831	4.5614
RIL-44	3.7994	1.9063	2.3812	2.6956	2.9851	2.0128	2.2041	2.4007
RIL-45	3.2047	2.7085	3.0849	2.9994	3.4324	2.1790	2.5379	2.7164
RIL-46	4.0147	2.2327	2.2064	2.8180	3.4500	2.2110	2.6012	2.7541
RIL-47	2.0698	1.4385	1.3913	1.6332	2.2591	1.2681	1.8560	1.7944
RIL-48	3.2104	2.0574	2.4205	2.5628	3.2286	1.9226	2.0810	2.4107
RIL-49	3.7966	2.1311	3.1191	3.0156	4.1876	2.3633	2.1293	2.8934
RIL-50	2.7798	1.6554	1.7917	2.0756	2.1620	1.5457	1.3597	1.6891
RIL-51	2.7525	1.5534	1.3996	1.9018	2.0256	1.5874	1.7400	1.7843
RIL-52	4.7797	3.4173	3.0059	3.7343	4.3428	2.8730	3.0813	3.4323
RIL-53	4.9595	3.8867	4.2604	4.3688	2.5131	2.0098	2.1821	2.2350
RIL-54	4.4667	2.7575	3.1144	3.4462	3.7276	2.5891	2.7505	3.0224
RIL-55	3.0891	2.9886	3.1613	3.0797	3.3942	2.4778	2.7693	2.8804
RIL-56	0.9691	0.9045	0.9296	0.9344	0.8740	0.5778	0.6392	0.6970
RIL-57	5.5017	2.7604	3.4481	3.9034	4.3537	2.9356	3.2147	3.5014
RIL-58	4.4485	3.7597	4.2821	4.1634	2.7138	1.7228	2.0065	2.1477
RIL-59	6.3840	3.5503	3.5085	4.4809	4.8465	3.7943	4.0387	4.2265
RIL-60	4.5866	3.1875	3.0830	3.6190	4.5616	3.0177	3.2365	3.6053
RIL-61	5.0683	3.2481	3.8213	4.0459	4.3104	3.4472	3.7426	3.8334
RIL-62	4.6797	2.6268	3.8446	3.7170	4.7903	2.9573	3.3400	3.6959
RIL-63	4.1537	2.4735	2.6773	3.1015	3.6192	2.1553	2.3327	2.7024
RIL-64	1.3691	0.7727	0.6962	0.9460	1.5367	0.8672	0.7814	1.0618
RIL-65	2.6664	1.9063	1.6769	2.0832	2.1247	1.5191	1.3362	1.6600
RIL-66	0.7332	0.5746	0.6298	0.6459	0.3148	0.2081	0.2302	0.2510
RIL-67	1.6269	1.0755	1.1898	1.2974	1.3859	0.9345	1.0233	1.1145
RIL-68	5.9472	4.0101	4.3913	4.7829	5.9952	3.8059	4.4328	4.7446
RIL-69	3.4075	2.1632	2.5195	2.6967	2.8728	2.2491	2.3940	2.5053
RIL-70	5.1496	4.0316	4.2913	4.4908	5.6029	3.7066	3.9753	4.4283
RIL-71	4.4268	2.9286	3.1409	3.4988	3.4543	2.7625	2.9993	3.0720
RIL-72	5.0379	4.0289	4.3743	4.4803	5.5040	4.1017	4.6750	4.7602
RIL-73	4.5542	3.0708	3.3628	3.6626	5.5160	3.4099	3.7317	4.2192
RIL-74	4.9256	3.1269	3.6419	3.8982	4.5886	2.7569	3.0164	3.4540
RIL-75	4.4175	3.4585	3.6813	3.8524	3.5782	3.2801	3.4993	3.4525
RIL-76	5.8915	3.8975	4.1801	4.6564	6.4921	3.4489	3.8547	4.5986
RIL-77	2.6338	2.1063	2.2868	2.3423	3.4743	2.0508	2.1956	2.5735
RIL-78	2.7533	2.0518	2.3386	2.3812	2.3965	2.2108	2.2502	2.2858
RIL-79	5.2429	3.2411	3.5469	4.0103	2.3051	2.0410	2.1152	2.1538
RIL-80	5.8915	3.5397	3.8729	4.4347	5.4157	3.1968	4.0493	4.2206
RIL-81	0.8670	0.7948	0.8479	0.8365	1.1423	0.7052	0.7965	0.8813
RIL-82	6.1435	3.2637	3.6477	4.3516	4.7358	4.5818	4.8465	4.7213
RIL-83	3.4562	2.0401	2.1841	2.5601	2.3071	2.1533	2.2133	2.2246
RIL-84	1.4103	1.3010	1.3242	1.3452	1.8583	0.9324	1.1647	1.3184
RIL-85	4.7409	4.1977	4.3503	4.4296	4.5344	3.8324	4.3648	4.2439
RIL-86	5.4043	3.1900	4.0407	4.2117	5.8771	3.2685	3.2300	4.1252

RIL-87	6.1532	3.7987	4.2903	4.7474	5.5333	3.8454	3.7193	4.3660
RIL-88	1.0871	1.0518	1.1125	1.0838	1.2756	0.8175	0.9618	1.0183
RIL-89	4.4276	4.1324	4.2475	4.2692	5.2877	2.9681	4.3440	4.1999
RIL-90	3.0304	1.8708	2.1129	2.3381	3.2150	1.9145	2.0722	2.4006
RIL-91	0.7970	0.7711	0.8157	0.7946	1.5519	0.8758	0.7891	1.0723
RIL-92	3.4686	3.2373	3.3274	3.3444	4.0161	2.8713	2.5257	3.1377
RIL-93	5.2992	2.6588	3.3212	3.7597	4.0228	3.1526	3.4557	3.5437
RIL-94	4.9152	4.1542	4.7313	4.6002	5.6600	3.7444	4.0159	4.4734
RIL-95	5.1491	2.8636	2.8299	3.6142	3.5835	2.8658	3.1114	3.1869
RIL-96	2.4209	1.6825	1.6273	1.9102	2.5014	1.7374	1.8457	2.0282
RIL-97	5.2668	3.3753	3.9710	4.2044	4.5123	3.2941	3.6816	3.8294
RIL-98	6.1051	3.4269	5.0156	4.8492	5.9451	3.9304	4.3479	4.7411
RIL-99	5.8746	3.4983	3.7864	4.3864	5.9398	4.0051	4.3859	4.7769
RIL-100	6.1265	3.4575	3.1151	4.2330	2.6995	1.7137	1.9960	2.1364
RIL-101	4.7716	3.4115	3.0008	3.7280	4.1408	3.2418	3.4506	3.6111
RIL-102	3.2006	2.5083	2.7494	2.8194	3.4197	2.2623	2.4263	2.7028
RIL-103	2.3607	1.5618	1.6750	1.8658	1.9716	1.5768	1.7119	1.7534
RIL-104	2.5055	2.0037	2.1755	2.2282	2.6206	1.6178	1.8272	2.0219
RIL-105	6.2323	4.2023	4.6018	5.0121	5.9141	3.7544	4.3728	4.6804
RIL-106	4.0618	2.5786	3.0033	3.2145	4.0949	2.8442	3.0215	3.3202
RIL-107	1.3994	1.0956	1.1662	1.2204	1.4933	1.0902	1.2184	1.2673
RIL-108	2.7087	1.7920	1.9219	2.1409	2.7000	1.7850	1.9746	2.1532
RIL-109	4.2777	3.4210	3.7142	3.8043	2.5632	1.7283	1.8926	2.0613
RIL-110	5.5333	4.1236	4.6999	4.7856	5.9044	3.7483	4.3656	4.6728
RIL-111	1.7169	1.0613	1.1615	1.3132	1.4020	1.0976	1.1683	1.2227
RIL-112	2.0478	1.2304	1.3462	1.5414	1.9809	1.3105	1.4055	1.5656
RIL-113	2.4378	2.2346	2.3840	2.3522	2.6512	2.1203	2.3020	2.3578
RIL-114	3.8944	2.0689	2.3123	2.7586	2.7763	2.0690	2.3582	2.4012
RIL-115	3.5910	2.1197	2.2693	2.6600	3.4844	2.1540	2.3572	2.6652
RIL-116	4.3034	3.9698	4.0407	4.1047	5.2600	3.1603	3.4577	3.9593
RIL-117	3.4252	3.0327	3.1430	3.2003	3.2838	3.0101	3.2114	3.1684
RIL-118	4.3012	2.5389	3.2159	3.3520	4.8010	2.5505	2.8506	3.4007
RIL-119	5.5624	3.4340	3.8784	4.2916	5.8130	3.4313	3.6735	4.3059
RIL-120	4.3592	4.2174	4.4611	4.3459	4.8541	4.4778	4.5577	4.6299
RIL-121	1.9840	1.8517	1.9033	1.9130	1.7988	1.5927	1.6506	1.6807
RIL-122	3.6125	2.2302	2.5188	2.7872	3.6263	2.1405	2.7114	2.8261
RIL-123	2.7871	2.6965	2.8523	2.7786	3.7502	2.3152	2.6148	2.8934
RIL-124	3.3658	3.1415	3.2289	3.2454	3.1521	3.0496	3.2258	3.1425
RIL-125	6.1856	3.1035	3.8767	4.3886	4.1286	3.8533	3.9606	3.9808
RIL-126	3.9958	3.3771	3.8463	3.7398	5.0576	2.5376	3.1698	3.5883
RIL-127	5.9968	3.3350	3.2957	4.2092	4.3581	3.6833	4.1951	4.0788
RIL-128	5.7698	4.0098	3.8783	4.5526	7.0354	3.9126	3.8665	4.9382
RIL-129	2.1628	1.3861	1.6307	1.7266	2.1837	1.5176	1.4678	1.7231
RIL-130	6.1418	3.4475	5.0457	4.8783	4.7057	4.5527	4.8157	4.6914

RIL-131	2.9165	1.7368	1.8798	2.1777	1.8018	1.6817	1.7285	1.7374
RIL-132	6.0838	3.4335	3.0934	4.2036	5.7694	2.8947	3.6159	4.0933
RIL-133	3.0816	2.2032	1.9380	2.4076	2.4917	2.1059	2.3985	2.3320
RIL-134	1.9890	1.5588	1.7087	1.7522	3.0838	1.7150	1.6948	2.1645
RIL-135	2.7928	1.8476	1.9815	2.2073	2.6884	1.8683	1.8070	2.1212
RIL-136	5.2272	4.1804	4.5387	4.6488	5.4243	3.4763	4.0897	4.3301
RIL-137	3.3196	1.6656	2.0805	2.3552	3.1800	1.7850	2.6125	2.5258
RIL-138	4.7726	4.0336	4.5940	4.4667	5.4888	3.5176	4.1384	4.3816
RIL-139	6.0449	3.3617	3.3222	4.2429	6.0726	3.4087	4.9889	4.8234
RIL-140	4.0225	2.7955	2.7038	3.1739	3.9815	2.3710	2.5662	2.9729
RIL-141	0.8190	0.5249	0.6175	0.6538	0.9657	0.5450	0.4910	0.6673
RIL-142	0.9286	0.5212	0.7628	0.7375	0.3208	0.2293	0.2017	0.2506
RIL-143	3.3508	1.9954	2.1597	2.5019	2.6180	2.0517	2.2490	2.3063
RIL-144	0.9742	0.5498	0.4953	0.6731	0.6057	0.4007	0.4298	0.4787
RIL-145	4.2277	3.0226	2.6588	3.3030	3.0892	2.4705	2.6823	2.7473
RIL-146	0.8362	0.5638	0.6174	0.6725	0.4910	0.3410	0.3623	0.3981
RIL-147	0.8014	0.5087	0.5925	0.6342	0.5118	0.3737	0.4176	0.4344
RIL-148	1.7811	1.3944	1.4843	1.5533	2.1874	1.4461	1.5998	1.7444
RIL-149	2.9892	1.9775	2.1209	2.3625	2.9827	2.0112	2.2024	2.3988
RIL-150	0.9598	0.7675	0.8333	0.8536	0.6299	0.3999	0.4658	0.4985
RIL-151	4.3719	3.2580	3.7134	3.7811	3.7199	2.9123	3.1000	3.2441
RIL-152	1.0560	0.6528	0.7144	0.8077	0.9078	0.6005	0.6441	0.7175
RIL-153	1.9136	1.1497	1.2579	1.4404	1.6155	1.2920	1.4027	1.4368
RIL-154	2.5332	2.3221	2.4773	2.4442	3.3873	2.0912	2.3618	2.6134
RIL-155	1.0080	0.5355	0.5985	0.7140	0.7318	0.4319	0.4624	0.5421
RIL-156	5.7546	3.3968	3.6366	4.2627	3.6954	3.4089	3.4698	3.5247
RIL-157	1.0565	0.9746	0.9920	1.0077	0.8166	0.7231	0.7494	0.7630
RIL-158	0.7814	0.6919	0.7171	0.7301	0.4357	0.2572	0.3258	0.3396
RIL-159	1.3383	0.7900	1.0007	1.0430	1.1542	0.7125	0.8047	0.8905
RIL-160	3.4332	2.1195	2.3938	2.6488	4.1357	4.0012	4.2323	4.1231
RIL-161	4.8054	4.6492	4.9178	4.7908	4.3343	4.0453	4.1579	4.1792
RIL-162	4.0042	3.7372	3.8413	3.8609	3.3130	2.4185	2.7030	2.8115
RIL-163	4.2822	2.8310	3.1318	3.4150	3.7500	2.4792	2.7426	2.9906
RIL-164	3.3007	2.2256	2.4372	2.6545	2.9465	1.9867	2.1756	2.3696
RIL-165	1.1830	0.7510	0.8747	0.9362	0.4896	0.3108	0.3620	0.3875
RIL-166	0.7866	0.6158	0.6555	0.6860	0.3889	0.3763	0.3980	0.3877
RIL-167	3.4941	2.3116	2.4791	2.7616	2.7827	2.5971	2.6694	2.6831
RIL-168	0.7924	0.6337	0.6880	0.7047	0.9854	0.4944	0.6176	0.6991
RIL-169	5.0306	3.1056	3.5075	3.8812	3.3997	2.8733	3.2725	3.1819
RIL-170	0.6396	0.6188	0.6546	0.6377	0.6029	0.3353	0.3314	0.4232
RIL-171	0.9894	0.9234	0.9491	0.9540	0.6677	0.4640	0.4488	0.5268
RIL-172	0.9792	0.4913	0.6137	0.6947	0.6488	0.4158	0.4892	0.5179
RIL-173	1.8756	1.5852	1.8055	1.7554	2.0190	1.1333	1.6587	1.6037
RIL-174	2.0239	1.1255	1.1123	1.4206	2.3280	1.1680	1.4590	1.6517

RIL-175	0.8141	0.5658	0.5472	0.6423	0.3166	0.2676	0.3048	0.2963
RIL-176	2.5830	1.6554	1.9475	2.0620	2.7657	1.5381	1.5200	1.9413
RIL-177	3.6610	2.0550	3.0076	2.9079	3.5707	2.4815	2.4001	2.8174
RIL-178	3.1685	1.8868	2.0422	2.3658	2.8044	2.1955	2.3370	2.4457
